# Multiparametric MRI in Biopsy Guidance for Prostate Cancer: Fusion-Guided

**DOI:** 10.1155/2014/439171

**Published:** 2014-07-13

**Authors:** Jason T. Rothwax, Arvin K. George, Bradford J. Wood, Peter A. Pinto

**Affiliations:** ^1^Urologic Oncology Branch, National Cancer Institute, National Institutes of Health, Bethesda, MD 20892-1210, USA; ^2^Center for Interventional Oncology, National Cancer Institute, National Institutes of Health, Bethesda, MD 20892-1210, USA

## Abstract

Prostate cancer (PCa) is the most common solid-organ malignancy among American men and the second most deadly. Current guidelines recommend a 12-core systematic biopsy following the finding of an elevated serum prostate-specific antigen (PSA). However, this strategy fails to detect an unacceptably high percentage of clinically significant cancers, leading researchers to develop new, innovative methods to improve the effectiveness of prostate biopsies. Multiparametric-MRI (MP-MRI) has emerged as a promising instrument in identifying suspicious regions within the prostate that require special attention on subsequent biopsy. Fusion platforms, which incorporate the MP-MRI into the biopsy itself and provide active targets within real-time imaging, have shown encouraging results in improving the detection rate of significant cancer. Broader applications of this technology, including MRI-guided focal therapy for prostate cancer, are in early phase trials.

## 1. Introduction

This year, prostate cancer (PCa) will comprise an estimated 27% of new cancer diagnoses in American men, making it the most common solid-organ malignancy among this population [1]. It is currently most often diagnosed on random systematic ultrasound-guided biopsies prompted by elevated serum PSA levels. Over the last three decades, efforts have been made to improve PCa detection using prebiopsy imaging to locate targets of suspicion for guided biopsy rather than to rely on random sampling. Most recently, the introduction of multiparametric-MRI (MP-MRI), which typically incorporates T2-weighted (T2W), diffusion-weighted (DW), and dynamic contrast enhanced (DCE) modalities, as well as MR spectroscopy (MRS) in some centers, has further enhanced the potential of imaging in the diagnosis and staging of PCa. The technology was first incorporated into biopsies via the in-bore technique, in which radiologists sample the prostate while the patient is undergoing MRI. This technique, while accurate, may be costly, more time-consuming, and cumbersome for both patient and physician. Fusion-guided biopsies, which incorporate this MR-imaging into the traditional ultrasound procedure, have been introduced as the next step in the evolution of prostate cancer detection. Several different methods have been developed and tested, with encouraging outcomes.

## 2. Background

Transrectal ultrasound (TRUS) has been the traditional means of imaging the prostate and today remains the most common modality. It is a low-cost, practical tool for live imaging during procedures in which the boundaries of the prostate and its adjacent structures must be visualized, including biopsies and brachytherapy. What TRUS fails to provide, however, is information that pertains to the composition of the prostate tissue itself, whether it is benign or malignant and, if malignant tissue is present, whether it has spread beyond the prostate capsule [2]. As biopsies are currently guided by ultrasound alone, standard-of-care practice relies on a 12-core template that randomly samples different regions of the prostate gland. Unfortunately, this method detects prostate cancer just 27–44% of the time [3–5] and has limitations including operator variability and failure to adequately sample the anterior-most portions of the prostate [6]. This leads to 20% false negatives and a 30–45% risk of pathological upstaging [7, 8] or downstaging [9] in men classified as having low-risk disease who proceed to radical prostatectomy. Additionally, without target resampling of suspicious areas, the utility and diagnostic yield of 12-core biopsy diminish with additional random rebiopsy [10]. For these reasons, alternative modalities for imaging the prostate have been investigated.

Improvements in MRI technology over the last several decades, however, as well as the integration of anatomical/structural imaging and functional/molecular imaging have led not only to renewed exploration into the potential for MRI in staging biopsy-confirmed prostate cancer but also to detecting cancer* prior* to biopsy and, perhaps, changing the way we think about initially approaching patients who present with an elevated PSA.

### 2.1. Utility of MP-MRI in Prostate Cancer Detection

The combination of anatomic imaging (T1- and T2-weighted) and functional imaging (diffusion-weighted, dynamic contrast-enhanced, and spectroscopic) has led to a significant improvement in the accuracy and utility of MR imaging of the prostate. With the combination of T2-weighted (T2W), diffusion-weighted imaging (DWI), dynamic contrast-enhanced (DCE), and magnetic resonance spectroscopy (MRS), the radiologist is now able to generate a more useful interpretation to supplement clinical findings.

T2W imaging delineates the prostate anatomy with high-resolution images, while DWI takes advantage of the varying diffusion of water molecules between normal tissue types and different pathological processes to produce apparent diffusion coefficients (ADC) that provide contrast between malignant and benign tissues. DCE involves the administration of contrast intravenously, followed by a rapid series of scans and perfusion measurements. Normal benign prostate tissue is perfused at a different rate than that of cancerous tissue, providing the radiologist with more detail about the nature of the tissue imaged. Finally, MRS allows for measurements of metabolic activity in the prostate. Concentrations of choline and creatine increase in correlation with tumor volume and grade, while citrate concentration decreases, again offering the radiologist a better understanding of the imaged tissue.

The significant overlap between benign and malignant tissue identified on any individual sequence justifies the multiparametric approach. The greater the number of parameters read by the radiologist, the greater the accuracy for detecting prostate cancer [2, 11, 12].

## 3. Fusion Guided Biopsies

While prostate cancer detection and staging on MP-MRI alone may be an option in the future, currently lesions identified on MP-MRI are confirmed with prostate biopsy. Detection of cancer on biopsy could be improved by targeting these lesions, however, and researchers have sought out the best way to utilize the MP-MRI during biopsy. Three techniques for using MR images to improve prostate biopsies have been explored thus far: in-bore, cognitive registration, and software registration-based MRI-TRUS fusion.

### 3.1. In-Bore MR-Guided Biopsy

In-bore MR-guided biopsies were the first targeted biopsies performed using MRI-identified lesions. After an initial diagnostic MP-MRI, the patient undergoes a biopsy within the bore of the magnet at a later date. He is placed prone into the scanner and core needles are used to sample the visible lesions using MR fluoroscopy, a rapid MR technique. Successive images are acquired to confirm needle placement and biopsy locations are documented precisely. This method can be performed via a transrectal approach, as is most common, or, alternatively, can be transperineal or transgluteal [13, 14]. The latter methods are less popular as they are considered more invasive and, in some cases, require sedation or even general anesthesia.

The major advantage of the in-bore technique is that it offers the most accurate targeting of the MRI-identified lesions and reduced detection of insignificant tumors. Additionally, only a few targeted cores are taken. However, there exist several drawbacks. First, this method requires significant additional training for the physician. Secondly, most MR-guided biopsies can take 60–90 minutes to be completed, depending on the operator's experience [15]. This lengthy imaging time and the necessity of two MR sessions result in increased costs associated with this method. The cost effectiveness has been debated, and while some models state it to be nearly equivalent to that of traditional TRUS-guided biopsy, others question this conclusion [16, 17]. However, the cancer detection rates appear to be equivalent or improved, especially with regard to detection of potentially lethal PCa. In a systematic review by Overduin and colleagues, results of in-bore biopsies showed PCa detection rates ranging from 8 to 59% (median 42%). The majority of tumors detected by in-bore biopsy were clinically significant (81–93%) [18].

### 3.2. Cognitive Registration

Cognitive registration is the most basic method to understand and adopt. Suspicious lesions are identified on prebiopsy MP-MRI and targeted on TRUS by approximating location following review of the imaging. This method is advantageous in that it requires no additional training of the operator with unfamiliar software or hardware upgrades. The MRI of the prostate and its presentation on TRUS are mentally coregistered by the physician performing the biopsy. The major shortcoming, however, is that the mental map method relies heavily on practitioner experience and therefore confers a great deal of interoperator variability and potential inaccuracies. It has been performed successfully by experts, but the ability to transfer the skill to urologists in the community has not been established. Biopsies conducted with cognitive registration guidance demonstrated increased detection rates and accuracy compared with systematic biopsies [19, 20]. Puech et al. showed a 10% increase in detection of prostate cancer in targets identified on MRI and a 15% increase in high-grade disease, compared with systematic biopsies [21].

### 3.3. Software-Based Registration Platforms

**Figure 1:**
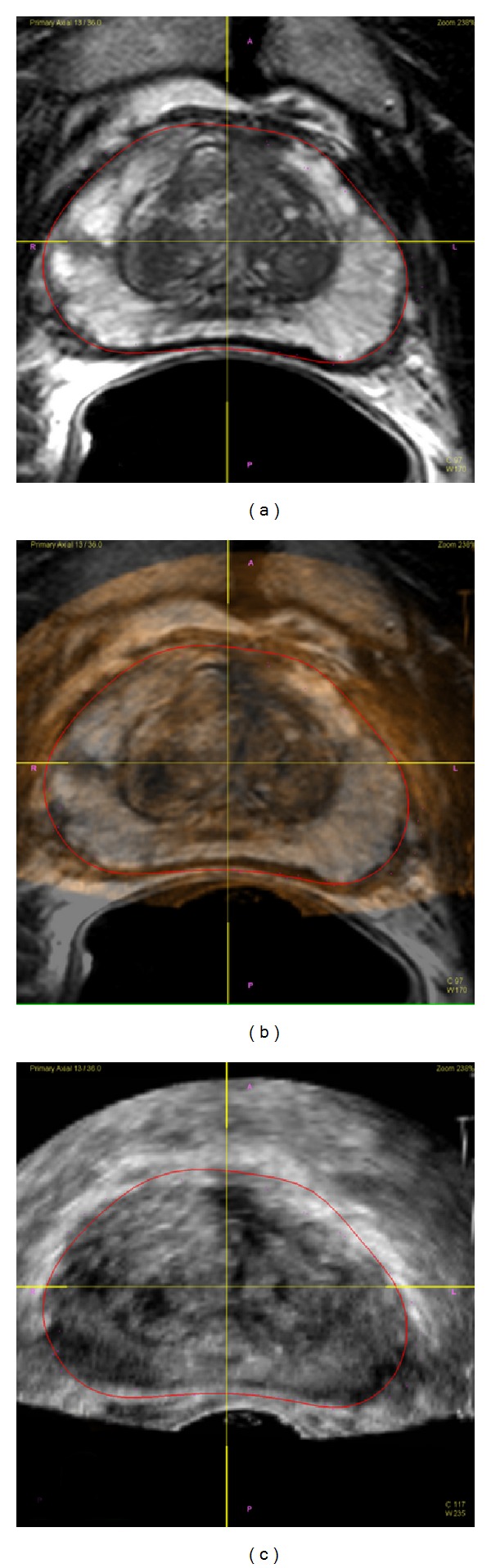
(a) Axial-view MRI of prostate. (b) MRI overlay on axial TRUS. (c) Axial-view TRUS of prostate.

Software-based registration platforms like the MRI-TRUS fusion biopsy systems were conceived and developed in an attempt to offer a low-cost, accurate alternative to in-bore prostate biopsies that can be performed by any urologist in an office setting with minimal additional training. Before being adapted for use in prostate biopsies, the technology was initially developed for central nervous system applications [22] and subsequently applied in prostate brachytherapy [23]. Again, prior to biopsy, the patient undergoes an MP-MRI from which suspicious lesions are identified. Additionally, the contour of the prostate is outlined or “segmented,” and this information is sent to the fusion software platform. At the time of biopsy, a typical 2D TRUS is obtained. The 2D image of the prostate is then reconstructed into a 3D model by the computer platform, which is similarly segmented. The platform then overlays the TRUS image with the MRI, thereby “fusing” the contours of the prostate allowing the physician to target lesions seen only on the MP-MRI at the corresponding location within the real-time sonographic image Figure 1.

MRI-TRUS fusion guided biopsies have demonstrated detection rates superior to systematic biopsies [24]. This was especially true for higher-grade disease [25]. In a well-designed study by Siddiqui et al., in which patients underwent both MRI-TRUS fusion guided biopsies and systematic 12-core biopsies in the same session, fusion-guided biopsy upgraded and detected PCa of higher Gleason score in 32% of patients compared with traditional 12-core biopsy alone [26]. These results have been externally validated in other centers [27].

The accuracy of these targeted biopsies relies on precise registration of the prostate models, which is made difficult by the deformation of the prostate during the MRI (due to presence of endorectal coil) and the TRUS (due to variable pressures applied by a mobile probe). To address this issue, researchers have applied elastic registration, accounting for deformation of the gland on the initial MR image in order to match the real-time 3D computer-assisted construction of the TRUS image [28, 29]. This is in contrast to a rigid model, which would align the two models by simple rotation and magnification, requiring that they are identical on MR and TRUS. The ideal registration system would constantly update the matching contours of the two models as the probe is moved, keeping up with changes in shape and position. Current systems sometimes require the practitioner to make minor adjustments during the procedure. Comparing cognitive registration models with software-based fusion platforms, Delongchamps et al. found that cognitive registration (cognitive registration) resulted in no improvement in cancer detection over systematic biopsies (*P* = 0.66), while both rigid and elastic registrations lead to greater detection of significant prostate cancer and required fewer cores [30].

The final component of the fusion technique involves live guidance of the biopsy, known as “tracking.” To accomplish this, several different approaches have been researched leading to a number of devices approved by the FDA. These include external magnetic field generators, real-time 3D TRUS, and angle-sensing encoders in robotic arms.

#### 3.3.1. External Magnetic Field Generators

Developed entirely at the National Cancer Institute and in collaboration with scientists from Philips Research North American, the UroNav system (Philips-Invivo, Gainesville, FL) has the lengthiest period of clinical testing. For tracking, this system relies on an external magnetic field generator to create a localized positioning system. This is accomplished by attaching a small tracker to a traditional TRUS probe which communicates with the external generator and depicts the position of the probe relative to the prostate in real-time. The US image is acquired via manual sweep from base to apex using a freehand US probe. Because this device is familiar to urologists, the learning curve is relatively short. While overall detection rates with this system have been shown to be nearly equivalent to those of systematic biopsies (80 versus 81%), the addition of targeted cores increased the detection rates of intermediate and high risk cancers, with 32% of patients upgraded after targeted biopsy [31]. More specifically, this system detected cancer in nearly 90% of cases in whom a highly suspicious lesion was identified on MRI, and targeted cores were twice as likely to contain cancer as systematic cores [32]. The HI-RVS (Hitachi Real-time Virtual Sonography) system (Hitachi Medical Systems, Zug, CH) also uses an external magnetic field generator to track the biopsy and is integrated with an existing ultrasound probe. It can perform transperineal biopsies and uses a real-time biplanar TRUS for US image acquisition. A transrectal approach is currently under development [33].

#### 3.3.2. 3D TRUS Image Registration

The Urostation device (Koelis, La Tronche, France) has been shown to be highly accurate in a preclinical study [34]. It is similar to the UroNav device in that it relies on free-hand TRUS probe technique—again offering a short learning curve but also the potential for human error. It differs, however, in several manners. The system is dependent on the use of a compatible 3D TRUS probe, made by a limited number of companies and not typically used by urologists in the community. To track biopsies, it relies upon image registration alone rather than any external device. A reference model acquired at the beginning of a biopsy session is a combination of three registrations: first a rigid landmark-based registration, followed by a multiple point-based rigid registration, and finally an algorithmic, elastic, and 3D organ-based registration. This model is produced by relaying to the workstation the data acquired with the 3D probe. A biopsy is then performed, guided by the 3D TRUS probe and, after each core is obtained, the needle is left in place for several moments while another image is acquired and relayed to the workstation, identifying the location of the needle in the latest biopsy sample. The fact that the confirmation of needle location is made just* after* each core acquisition leads to potential targeting error. In one study, cancer was detected using this device in 48% of men with at least one prior negative biopsy and in 83% of those with highest suspicion on MRI [35].

#### 3.3.3. Angle-Sensing Encoders

A different approach was taken by the developers of the Artemis Device (Eigen, Grass Valley, CA). Rather than employing a magnetic field, a mechanical arm holds the TRUS transducer, allowing for angle-sensing encoders in each joint of the arm to track the position of the needle and probe. This robotic tracking technology offers superior accuracy and the ability to completely immobilize the probe from target acquisition to firing of the needle. The machine itself, however, is bulkier than the freehand devices and requires additional training for operation. The US image is acquired via manual rotation of the robotic arm along a fixed axis.

Early studies with this system at UCLA on men on active surveillance and on those with prior negative biopsies found cancer in 53% of men overall and in 94% of those with highest MRI suspicion. Targeted biopsies were three times more likely to identify cancer than systematic biopsies (20.8% versus 7.3%) [24]. Researchers at UCLA have also shown that this technology is particularly useful in repeat biopsy of specific cancer-containing sites. Data published by Sonn et al. demonstrated successful rebiopsy of 74 prior positive sites in men on active surveillance, dependent on the length of cancer on the initial biopsy and whether the site was associated with a visible lesion on MRI. 71% of sites contained cancer on rebiopsy when the initial cancer core length was ≥4 mm. Additionally, when cancerous sites were found in an MRI target, cancer was found on 61% of repeat biopsies versus 29% of those from systematic sites [36].

The BioJet system (GEOSCAN, Lakewood Ranch, FL) also uses a mechanical arm with encoders. This platform uses rigid registration and the US image is acquired with a manual sweep in sagittal position. This device allows for both transrectal and transperineal biopsies.

## 4. Growing Evidence of Utility

Evidence of a positive impact on cancer detection has been forthcoming; one recent systematic review by Moore et al. found that MRI-guided biopsies perform at the same level if not better than that of standard-of-care, ultrasound-only biopsies in identifying significant prostate cancer [37]. They further point out that this is accomplished using fewer cores, resulting in fewer complications and fewer findings of insignificant cancer, which is traditionally overtreated.

Furthermore, there are several common situations in which the application of MP-MRI guided biopsies would be particularly useful. Patients with negative systematic biopsies whose PSA continues to rise represent a population in which there exists special utility in MRI-guided biopsies [38, 39]. Studies have shown high positive diagnostic rates of PCa in these patients, from 34% to 52%, when an MP-MRI is employed and suspicious lesions are targeted [40, 41]. Several studies point out that the number of prior negative biopsy sessions was not predictive of cancer detection on follow-up MRI-guided biopsy, suggesting that early imaging in the course of PSA monitoring can be useful without concern for diminished sensitivity [42].

MP-MRI can also play a role in patients with no prior biopsies at all. A study by Park et al. showed that, compared with men undergoing systematic biopsy, those undergoing biopsy with initial MP-MRI guidance demonstrated a higher rate of cancer (29.5% versus 9.8%) and higher rate of positive cores (9.9% versus 2.5%) [20].

The prostate is much larger than the dozen or so cores sampled in a systematic biopsy, and it is widely accepted that there are regions of the gland, including the central and anterior portions, that are commonly undersampled and in which tumors cannot be detected on digital rectal examination [43]. MR-guided biopsies can be particularly useful in indicating to the physician the importance in sampling these regions, as one study reported that anteriorly located cancerous lesions were missed in up to 46% of 12-core systematic biopsies [44]. Additionally, Nix et al. demonstrated that targeted biopsies detected more cancers in the very distal apical prostate than systematic biopsies and found higher grade disease [45], while Diaz and colleagues found that MR-guided biopsies increased yield in enlarged prostates which tended to be undersampled [46].

## 5. Barriers and Limitations

There are certain limitations that have thus far prohibited the widespread availability of MP-MRI for prostate cancer detection. There is an inherent cost in performing prostate MRIs and in training radiologists to interpret the different modalities and their combination. However, performing just T2W and DWI sequences alone without an endorectal coil, which could be done quickly and at low cost, would likely result in fewer biopsies which would balance the increased cost of an MRI [47]. Another obstacle for radiologists has been the lack of a uniform, superior method for interpretation. Scoring systems have been proposed, including the PI-RADS and Likert scales, and have garnered praise among genitourinary radiologists. However, these systems are not yet widely accepted, as evidence in support of one over another is lacking, which is possibly only due to the short time since they have been proposed. One study which compared the PI-RADS and Likert scales found similar performance among three radiologists for tumor localization in the peripheral zone and better performance using the Likert scale in the transitional zone [48].

Even if the use of MP-MRI is widely adopted, there are barriers prohibiting the widespread use of image-guided biopsy including further demonstration of benefit and cost effectiveness. Current AUA guidelines call for additional data to conclusively prove that targeted biopsies increase benefit and reduce harm before prostate imaging can be recommended as first line test for early detection of prostate cancer [49]. Lastly, cost has been cited as a prohibitive factor, yet Sonn and colleagues point out that while image-guided biopsy may seem more expensive, as it requires MRIs and new devices, the consequences of missed diagnoses on systematic biopsy or overtreatment due to the ambiguity of results must be taken into account when assessing cost effectiveness [40], and further studies are required.

## 6. Future Directions

The fusion technology developed to detect prostate cancer can potentially be applied to its treatment. Focal therapy, including cryoablation, high intensity focused ultrasound, radiofrequency, and laser ablation, is emerging as an alternative to active surveillance in young, otherwise healthy men with low-volume, low-grade prostate cancer. A major challenge of these therapy modalities is to limit tissue destruction to the lesions and to avoid damage to the urethral sphincter and neurovascular bundles [50]. MR-guidance has therefore been explored as an instrument for the application of focal therapy in a localized and controlled manner. In current trials, focal therapies are typically performed in-bore [51] and within small case series, the feasibility of noninvasive focal ablation techniques was demonstrated [52]. Like in-bore biopsies, however, these techniques require time and are costly due to necessary resources including MR-compatible equipment. The ability to perform this therapy in an office setting using an MRI-TRUS fusion system would have obvious benefits. With significant advancements in the technology, these fusion platforms may one day enable the diagnosis and treatment of low-volume, low-grade disease in a single MRI-TRUS session. The fusion systems and methods currently show promise for therapeutic applications and further studies are certainly warranted.
